# 84 Context Analysis for Scaling-Up a Community-Based Physical Activity Promotion Approach: a Comparative Case Study of 10 German Pilot Communities

**DOI:** 10.1093/eurpub/ckae114.155

**Published:** 2024-09-26

**Authors:** Leonie Birkholz, Philipp Weber, Lea Dippon, Natalie Helsper, Simone Kohler, Klaus Pfeifer, Alfred Rütten, Jana Semrau

**Affiliations:** Friedrich-Alexander-Universität Erlangen-Nürnberg, Erlangen, Deutschland; Friedrich-Alexander-Universität Erlangen-Nürnberg, Erlangen, Deutschland; Friedrich-Alexander-Universität Erlangen-Nürnberg, Erlangen, Deutschland; Friedrich-Alexander-Universität Erlangen-Nürnberg, Erlangen, Deutschland; Friedrich-Alexander-Universität Erlangen-Nürnberg, Erlangen, Deutschland; Friedrich-Alexander-Universität Erlangen-Nürnberg, Erlangen, Deutschland; Friedrich-Alexander-Universität Erlangen-Nürnberg, Erlangen, Deutschland; Friedrich-Alexander-Universität Erlangen-Nürnberg, Erlangen, Deutschland

## Abstract

**Purpose:**

To achieve an impact on public health through community-based physical activity promotion (CBPA), it is crucial to navigate the resources available to build up structures for health promotion and health equity. As communities reveal different contexts and therefore varying needs for external support, an in-depth understanding of the initial conditions for successful large-scale implementation is required. This study aims to fill this gap by (1) analyzing the initial contextual conditions in 10 German pilot communities for the scale-up of a CBPA approach and (2) evaluating variations based on potential external support needs.

**Methods:**

We examined 10 German pilot communities, varying in type, geographical area, readiness, and socioeconomic deprivation. Employing a comparative case study design, we conducted online focus groups following a semi-structured interview guide with key stakeholders in each community. These included political stakeholders, administrative representatives, experts, and ‘door openers’ with links to socially disadvantaged people. Qualitative content analysis was used to compare similarities and differences in the initial conditions for the following 9 key components “resources”, “political support”, “communication”, “competence/qualification”, “existing structures”, “offers”, “strategic planning”, “intersectoral cooperation” and “participation”. We also compared them with the optimal external support for communities, based on the reported need for external support for implementation.

**Results:**

10 focus group interviews, each lasting 90 minutes, were conducted with 6-12 participants at the beginning of the implementation process. Differences were found in the 9 key components analysed in terms of type and socioeconomic deprivation. Moreover, differences were identified in the key components of “resources”, “political support”, “intersectoral cooperation” and “participation”. It was found that the key components of support needs are interdependent, so that a lack of resources, for example, is likely to occur in the absence of political support.

**Conclusions:**

The results provide important insights into the impact of external support for scaling up a CBPA approach. Based on the identified support needs of the pilot communities, tailored support formats such as workshops, consultations and manuals could be developed. This could facilitate further scaling-up processes.

**Support/Funding Source:**

This research was funded by the GKV Alliance for Health, grant number Z2/5-01-20-16.

